# Growth, fatty, and amino acid profiles of the soil alga *Vischeria* sp. E71.10 (Eustigmatophyceae) under different cultivation conditions

**DOI:** 10.1007/s12223-020-00810-8

**Published:** 2020-07-21

**Authors:** Daniel Remias, Cecilia Nicoletti, Klaus Krennhuber, Bettina Möderndorfer, Linda Nedbalová, Lenka Procházková

**Affiliations:** 1 School of Engineering, University of Applied Sciences Upper Austria, 4600 Wels, Austria; 2 Department of Ecology, Faculty of Science, Charles University, 12843 Prague, Czech Republic

## Abstract

In this study, a unicellular soil alga isolated from farmland in Germany was surveyed. The investigation of the hypervariable molecular markers ITS1 rDNA and ITS2 rDNA identified strain E71.10 as conspecific with *Vischeria* sp. SAG 51.91 (Eustigmatophyceae). The culture was tested for biomass generation and for the yield of fatty acids and amino acids. The survey included four different culture conditions (conventional, elevated CO_2_, nitrogen depletion, or sodium chloride stress) at room temperature. The best yield of dry biomass was achieved applying 1% CO_2_, whereas nitrogen-free medium resulted into least growth. The fatty acid content peaked in nitrogen-free medium at 59% per dry mass. Eicosapentaenoic acid was the most abundant fatty acid in all treatments (except for nitrogen free), accounting for 10.44 to 16.72 g/100 g dry mass. The highest content of amino acids (20%) was achieved under conventional conditions. The results show that abiotic factors strongly influence to which extent metabolites are intracellularly stored and they confirm also for this yet undescribed strain of *Vischeria* that Eustigmatophyceae are promising candidates for biotechnology.

**Electronic supplementary material:**

The online version of this article (10.1007/s12223-020-00810-8) contains supplementary material, which is available to authorized users.

## Introduction

Eustigmatophyceae have experienced increasing attention in the last years, because they turned out to be biotechnologically promising in terms of biomass generation, production of lipids, pigments, and other compounds (Stoyneva-Gärtner et al. 2019). Li et al. (2012) screened several species and found high levels of β-carotene (up to 5.9% dry mass) and biomass production rates up to 9.8 g/L in bubble column photobioreactors. Gao et al. (2016) reported a lipid production of 0.28 g/L/day for *Vischeria stellata* and stressed that this alga can be a source for nutraceuticals or used for biodiesel production. Moreover, a large proportion of the oleaginous components are polyunsaturated fatty acids, and significant amounts of the valuable eicosapentaenoic acid (EPA) have been reported for some of these algae (Cepák et al. 2014; Wang et al. 2018).

In this study, a yet undescribed strain of a eustigmatophycean alga isolated from field soil was tested for its potential of fatty and amino acid production. Both compound classes are of biotechnological interest. The taxonomic position was evaluated by using four molecular markers (18S rDNA, *rbc*L, ITS1 rDNA, ITS2 rDNA) which placed this alga into the genus *Vischeria*. Furthermore, lipid production was optimized during cultivation by applying stress protocols such as nitrogen depletion and addition of sodium chloride. To our knowledge, this alga has never been analyzed biochemically and phylogenetically before. Here, we showed that the strain *Vischeria* sp. E71.10 exhibited high growth rates and abundantly accumulated fatty acids, particularly eicosapentaenoic acid.

## Material and methods

A eustigmatophycean microalga was isolated by W. Oesterreicher (Kitzbühel, Austria) from field soil close to Ahlum (Wolfenbüttel, Lower Saxony, Germany) in course of a study testing changes of soil algae biocoenosis depending on the extent of insecticides and fertilization (Sautthof and Oesterreicher 1994). The strain was deposited as E71.10 at the algae culture collection at the Institute of Botany, University of Innsbruck, however was not listed in the strain catalog (ASIB; Gärtner 1996). The cell morphology was observed by light microscopy (Nikon Eclipse 80i, objective Plan Apo VC 100 × 1.40, camera DS 5 M; Nikon Instruments, Amsterdam, Netherlands) using either differential interference contrast or fluorescence mode (filter em = 600 LP, ex = 480/40). Prior use, the strain was grown at 15 °C and approximately 40 to 50 μmol photons/m^2^/s (14 h light, 10 h darkness) provided by full spectrum fluorescence 18 W tubes (Narva BioVital 958, Plauen, Germany). Modified Triple Nitrate Bolt’s Basal Medium (‘3 N BBM’) was used according to the recipe of the CCCryo strain collection (Potsdam, Germany, http://cccryo.fraunhofer.de/sources/files/medien/BBM.pdf). For the generation of biomass, the cells were cultivated at room temperature in 1-L glass column bubble reactors aerated with compressed air at 0.6 mL/min. The fluorescence tube illumination (14 h/day) was approximately 220 μmol photons/m^2^/s. Growth was evaluated by measuring the absorbance of a 3-mL subsample at 750 nm with a spectrophotometer (Lange Xion 500, Germany). The pH was monitored with a WTW electrode (Xylem Instruments, Weilheim, Germany). In alternative setup, the Erlenmeyer flasks with 3 N BBM medium were put into a growth chamber enriched with constant 1% CO_2_ supply (Percival SE-41AR3, CLF PlantClimatics, Germany). Two stress treatments for enhancement of fatty acid content were performed either by a complete medium change to nitrogen-free BBM (‘-N BBM’) or by adding 4 g/L NaCl to 3 N BBM. For harvesting, the reactors were discharged and the cell concentrated by centrifugation (3000*g*, 15 min). The algal pellet was immediately frozen at − 80 °C and subsequently lyophilized at darkness for 48 h.

### Analytics

The fatty acid (FA) content was measured by gas chromatography (GC) using a modified transmethylation protocol of Welz et al. (1990): 5 mg of lyophilized algae powder was suspended and methylated with 5 mL of methanol/acetyl chloride (p.a. grade) with a volumetric ratio of 50:1 for 4 h at 60 °C. The reaction was stopped by slowly adding 2.5 mL of a potassium carbonate solution (p.a. grade, 60 g/L). The resulting fatty acid methyl esters were extracted by adding 2 mL of hexane (GC/MS grade) and shaking for 2 min. After phase separation, 1 mL of the supernatant phase, containing the methyl esters, was transferred in a 1.5-mL crimp vial and stored at − 18 °C until measurement. The hexane-extract was injected in a Thermo Trace 1300 GC (Thermo Scientific), equipped with an autosampler AS 1310 and an SSL injector and flame ionization detector (FID). The chromatographic conditions were as follows: injection volume 1 μL, injector temperature was set at 240 °C. Helium was used as carrier gas (constant flow) with 1.5 mL/min and a split flow at 30 mL/min. An Agilent J&W capillary column DB-23 60 m, 0.25 mm ID, and 0.25 μm film thickness were used for analytical separation. The oven temperature gradient was 0–3 min 130 °C; 6.5 °C/min to 170 °C. 2.8 °C/min to 214 °C and held for 12 min. Then, 3 °C/min to 240 °C and held for 15 min. The FID was set at a temperature of 280 °C, 450 mL/min air flow, 45 mL/min hydrogen flow, and nitrogen as make up gas at 40 mL/min. Data analysis was performed with Chromeleon V7.2 (Thermo Scientific). The calibrations were done with an external standard (Supelco Fame Mix C14 - C22, Eicosapentaenoic acid methyl ester, Sigma-Aldrich). Determination of amino acid (AA) profiles. For protein digestion, a modified acidic hydrolyzation was performed according to Fountoulakis and Lahm (1998): 10 mg lyophilized algae sample was hydrolyzed with 1 mL of hydrochloric acid (c = 6 mol/L, analytical grade) in a 1.5-mL tight screw cap glass vial for 24 h at 110 °C. The hydrolysate was diluted appropriately in deionized water and clarified by centrifugation at 25,000*g* for 10 min. The clear supernatant was transferred in 2-mL HPLC vials using a final dilution in a range from 20-fold to 60-fold and stored at 4 °C prior analysis. The amino acids were measured on an Agilent 1260 Infinity II HPLC preforming an OPA-3-MPA (o-phthaldialdehyde *3*-mercaptopropionic acid) and FMOC-CL (fluorenylmethyloxycarbonyl chloride) pre-column derivatization and subsequent fluorescence detection (Schuster 1988). The stationary phase was a Thermo ODS Hypersil 2 column 4.6 × 250 mm kept at 40 °C. The injection volume was 10 μL and the pump flow rate was set to 1.5 mL/min and consisted of mobile phases (A) sodium dihydrogen phosphate buffer (c = 0.04 mol/L, analytical grade) and (B) acetonitrile, methanol (both in analytical grade) and deionized water in volumetric ratio of 45:45:10. The applied linear gradient was 0–3 min 2% B, 18 min 72% B, 18.1–25 min 100% B, re-equilibration time 6 min. For the two-step column derivatization of the sample, the following three reagents have been applied: (1) borate-buffer: made of *ortho-*boric acid, *c* = 0.4 mol/L at pH 10.2, adjusted with aqueous potassium hydroxide solution (*c* = 10 mol/L), syringe filtered (0.45 μm polyamide); (2) OPA-3-MPA reagent (Sigma-Aldrich): from 10 mg OPA in 1-mL borate buffer adding 10 μL 3-MPA; and (3) FMOC-CL reagent (Merck, Darmstadt, Germany): 2.5 mg in 1 mL acetonitrile. All chemicals were analytical standard grade. The final solutions were stored at 4 °C and useable for 1 week. Prior to the column injection, the injector program according to Table 1 was applied in the sample vial for adding the fluorophores, where FMOC derivatization was done for proline and OPA for the other measured amino acids.

**Table 1:** Injector program for pre-column derivatization, according to OpenLAB CDS ChemStation Edition using an Agilent 1260 autosampler

Step	Action	Parameter and reagent
1	draw	2.5 μL borate buffer 0.4 mol/L pH 10.2
2	draw	1.0 μL sample
3	mix	3.0 μL in air at maximum speed 6×
4	wait	0.2 min
5	draw	0.0 μL water (needle wash)^a^
6	draw	0.5 μL OPA-3-MPA reagent
7	mix	3.5 μL in air at maximum speed 10×
8	draw	0.0 μL water (needle wash)^a^
9	draw	0.5 μL FMOC-CL reagent
10	mix	4.0 μL in air at maximum speed 10×
11	draw	0.0 μL acetonitrile (needle wash)^a^
12	draw	32 μL diluent (water)
13	mix	18 μL in air at maximum speed 8×
14	injection	of derivatized sample
15	wait	0.1 min

^a^Needle submersion step in deionized water for cleaning

The detection of amino acids was performed by HPLC using fluorescence detection at ex/em 340/450 nm for OPA-3-MPA and ex/em 266/305 nm for FMOC-CL, the later especially applied for proline. The calibration for amino acid quantification was performed by external standardization with the amino acid standard mixture AAS18 and further single analytical standards from Merck. Asparagine and glutamine were hydrolyzed into their acids and are thus not detectable. Data acquisition and analysis were performed with OpenLAB CDS ChemStation Edition software (Agilent Technologies). All analytical samples were determined in biological and analytical triplets. Mean values were calculated by the arithmetic mean, standard deviations were calculated presuming a normal distribution and resulting the coefficient of variation was shown. The yield was calculated as compound production rate per day of reactor life time.

### Molecular methods

Total genomic DNA of the strain E71.10 was extracted according to Procházková et al. (2018). The 18S small subunit ribosomal RNA gene (18S rDNA), internal transcribed spacer regions 1 and 2 (ITS1 rDNA, ITS2 rDNA), and ribulose-1,5-bisphosphate carboxylase/oxygenase large subunit (*rbc*L) gene regions were amplified from DNA isolates by polymerase chain reaction using existing primers (Table S1). Amplification and sequencing reactions for these markers were identical to those described by Procházková et al. (2018). Nuclear rDNA regions of ITS2 were identified using the web interface for hidden Markov model-based annotation (Keller et al. 2009) at the ITS2 database (Ankenbrand et al. 2015; http://its2.bioapps.biozentrum.uni-wuerzburg.de/). The sequence was then folded with 5.8S–LSU stem regions using the Mfold server accessible at http://mfold.rna.albany.edu/?q5mfold (Zuker 2003). A model of the secondary structure consistent with the specific features of nuclear rDNA ITS2 was selected: four helixes and U–U mismatch in helix II (Coleman 2007). For detecting compensatory base changes (CBCs), the ITS2 sequences were aligned based on a sequence-structure analysis (Schultz and Wolf 2009) using 4SALE (Seibel et al. 2006, 2008). The secondary structure of nuclear rDNA ITS2 was drawn using VARNA version 3.9 (Darty et al. 2009). The correlation between the CBC criterion and the biological species concept was introduced by Coleman (2000) and was statistically proven by Müller et al. (2007): if a CBC is found between two organisms classified within the same genus, then they are two different species with 93% probability. Presence of CBC was checked between the strain E71.10 and its closest relative strain SAG 51.91. The obtained sequences were submitted to the National Center for Biotechnology Information (NCBI) Nucleotide sequence database (accession numbers 18S rDNA: MN781105; ITS1 rDNA + 5.8S rDNA + ITS2 rDNA: MN781106; *rbc*L: MN781107).

## Results

### Morphological and molecular characterization

**Fig. 1:**
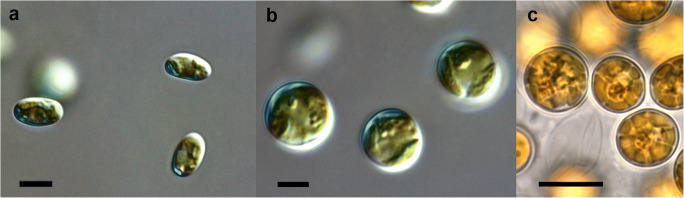
Light microscopy of *Vischeria* sp. E71.10. **a** Young cells elongate ellipsoidal, **b** typical mature cells almost spherical, with irregular cup-shaped chloroplast, **c** aged cells in nitrogen-depleted medium with orange intracellular pigmentation. Scale: 10 μm

Young cells of strain E71.10 were solitary elongate and more or less characteristically bean shaped (Fig. 1a); they became larger and changed to irregularly ellipsoidal to almost spherical appearance (Fig. 1b). Wall surfaces were smooth; bulges typically for some Eustigmatophyceae occurred rarely. At old stages, the alga turned from green to orange showing a prominent extraplastidal pyrenoid. A bi- or tri-layered cell wall was visible (Fig. 1c). Most of the cells were immotile in both liquid cultures and on agar, but flagellates occurred occasionally (Supplementary Video 1). The latter had two visible flagella, one of them very short. All cells had a single parietal chloroplast, cup-shaped with incisions or lobes; however, no eyespot was detected. Cell division by autosporangia was observed.

The 18S rDNA sequence of strain E71.10 was 100% identical with several strains assigned to three different genera *Eustigmatos*, *Vischeria*, and *Chloridella* (Supplementary Table S2). One of them was *Vischeria* sp. CAUP Q 202 (Yurchenko et al. 2016). For *rbc*L, strain E71.10 was closely related to *Vischeria helvetica* UTEX 49 (Yang et al. 2012). Moreover, the alga was almost identical for the hypervariable markers ITS1 and ITS2 rDNA with *Vischeria* sp. SAG 51.91, isolated from snow detritus in the Belianské Tatry Mountains (Slovakia) (Supplementary Table 2). ITS2 rRNA secondary structure of strain E71.10 and SAG 51.91 was compared; no CBC was found in the entire structure (Supplementary Fig. 1).

### Biomass, fatty, and amino acid yield

Table 2 shows the total harvested dry mass (DM) of *Vischeria* sp. E71.10, the daily dry mass production rate, and the total content of fatty acids, respectively, amino acids per dry mass for all four culture treatments. The by far best yield of dry biomass was achieved applying elevated CO_2_ (1%) in the atmosphere of the chamber in combination with 3 N BBM medium, whereas nitrogen-free medium resulted into least growth. The fatty acid content of approx. 42% for 3NBBM was outraged when exposing cells to nitrogen-free medium (-NBBM) resulting into almost 59% FAs per dry mass. On the other hand, -NBBM showed the least growth and biomass accumulation rates, succeeded by sodium chloride stress as the second least. The latter hardly changed the relative cellular FA and amino acid content compared to the standard treatment.

**Table 2:** Production rates of *Vischeria* sp. E71.10 under four different cultivation treatments showing the total harvested biomass (DM per reactor volume), daily biomass growth, total fatty acid content (TFA), and total amino acid content (TAA) per dry mass

Treatment	Biomass (DM) (g/L)	Productivity (mg DW/L/day)	TFA (g/100 g)	TAA (g/100 g)
3NBBM	1.31 ± 0.17	46.93 ± 5.90	23.48 ± 0.71	20.74 ± 2.02
3NBBM + 4 g/L NaCl	0.91 ± 0.04	32.61 ± 1.50	25.45 ± 0.66	20.43 ± 0.69
3NBBM + CO_2_	3.87 ± 0.15	193.55 ± 7.30	42.07 ± 2.28	9.88 ± 1.59
-NBBM	0.45 ± 0.08	15.95 ± 2.94	58.96 ± 1.76	5.63 ± 0.31

By means of GC, the abundance of fatty acids was compared among the four treatments (Table 3). The sizes of main fatty acids ranged from C14:0 to C20:4, and they were qualitatively similar between all treatment except the nitrogen-free exposure; in the latter case, C16:1 (palmitoleic acid) was particularly increased. In all other assays, C20:5 (eicosapentaenoic acid, EPA) was the most abundant fatty acid in a range of 10.4 to 16.7% of dry mass.

**Table 3:** Cellular content of principal fatty acids [g/100 g DM] in *Vischeria* sp. E71.70 after the four different treatments

Fatty acid class	Treatment			
	3NBBM	3NBBM + 4 g/L NaCl	3NBBM + CO_2_	-NBBM
C14:0	0.61 ± 0.06	0.62 ± 0.05	1.09 ± 0.11	2.50 ± 0.15
C16:0	4.33 ± 0.39	4.26 ± 0.21	5.41 ± 0.28	8.23 ± 0.44
C16:1	4.62 ± 0.32	5.15 ± 0.34	13.41 ± 1.02	25.90 ± 0.55
C18:0	0.43 ± 0.06	0.44 ± 0.05	0.31 ± 0.04	1.15 ± 0.09
C18:1	0.97 ± 0.09	0.96 ± 0.07	3.90 ± 0.23	6.91 ± 0.27
C18:2	1.77 ± 0.16	1.72 ± 0.10	1.00 ± 0.05	1.57 ± 0.13
C18:3	0.31 ± 0.01	0.32 ± 0.03	0.25 ± 0.01	0.59 ± 0.03
C20:5	10.44 ± 0.24	11.99 ± 0.66	16.72 ± 1.16	12.12 ± 0.48

The spectrum of cellular amino acids was measured by HPLC as shown in Table 4. A broad range of AAs was found, and the sodium chloride stress did hardly change the absolute level of about 20% per dry mass. However, during the fast growth under CO_2_ application, AA content decreased to approx. 10% and was even less (5.5%) during nitrogen depletion.

**Table 4:** Cellular content of amino acids [g/100 g DM] in *Vischeria* sp. E71.70 after the four different treatments

Amino acid	Treatment			
	3NBBM	3NBBM + 4 g/L NaCl	3NBBM + CO_2_	-NBBM
Asparagine	2.84 ± 0.28	2.78 ± 0.09	1.44 ± 0.33	0.79 ± 0.21
Glutamic acid	3.22 ± 0.32	3.13 ± 0.07	1.19 ± 0.20	0.82 ± 0.19
Serine	1.27 ± 0.14	1.23 ± 0.08	0.35 ± 0.06	0.57 ± 0.06
Glycine	1.78 ± 0.22	1.65 ± 0.09	0.69 ± 0.15	0.85 ± 0.23
Threonine	1.17 ± 0.09	1.08 ± 0.03	0.45 ± 0.07	n.d.
Arginine	3.44 ± 0.35	3.37 ± 0.10	1.47 ± 0.21	0.86 ± 0.04
Alanine	0.42 ± 0.07	0.34 ± 0.07	0.11 ± 0.02	n.d.
Tyrosine	0.78 ± 0.07	0.82 ± 0.09	0.44 ± 0.02	n.d.
Methionine	1.34 ± 0.11	1.28 ± 0.06	0.75 ± 0.13	n.d.
Phenylalanine	1.02 ± 0.15	1.14 ± 0.03	0.69 ± 0.10	0.42 ± 0.02
Isoleucine	0.72 ± 0.08	0.75 ± 0.01	0.49 ± 0.10	0.48 ± 0.03
Leucine	1.45 ± 0.63	1.78 ± 0.06	1.12 ± 0.17	0.40 ± 0.00
Lysine	1.29 ± 0.29	1.07 ± 0.03	0.69 ± 0.08	0.44 ± 0.01

*TAA* total amino acids, *n.d.* not detected

## Discussion

### Cell morphology and taxonomy

In Eustigmatophyceae, the 18S rDNA marker has a resolution useful for definition of the new clades (e.g., Goniochloridales) but is not suitable for species determination (Fawley et al. 2014). Instead, the use of a polyphasic approach including the more variable ITS2 rDNA marker is more reliable for defining species boundaries in microalgae (Kryvenda et al. 2018). The investigated strain E71.10 was almost identical for hypervariable markers ITS1 and ITS2 with strain SAG 51.91. At the LM level, strain E71.10 shared with SAG 51.91 vegetative cells without wall projections, bean shaped to spherical during cell aging, one single plastid per cell throughout the cell cycle; however, for the latter, the formation of zoospores was not observed (Kryvenda et al. 2018). In *Eustigmatos* and *Vischeria*, the flagellated stages possess a single emergent flagellum. In strain E71.10, two visible flagella were present, one of them very short. Using the key of Ettl and Gärtner (2014), the ellipsoidal to spherical smooth cell shape in E71.10 cells resembled *Pseudellipsoidion* (Neustupa and Němcová 2001)*.*

In the 18S rDNA phylogeny of Eustigmatophyceae, strains previously assigned to *Eustigmatos*, *Vischeria*, and *Chloridella* were recovered within a well-supported monophyletic clade, the ʿEustigmataceae Groupʾ sensu (Fawley et al. (2014). Based on the sequence-structure ITS2 rDNA phylogeny, this latter group was further distinguished into a ʿ*Vischeria* cladeʾ and a ʿ*Vischeria*-related cladeʾ (Kryvenda et al. 2018). Consequently, all taxa of the ʿ*Vischeria* cladeʾ should be transferred into the genus *Vischeria* (Kryvenda et al. 2018). Therefore, strain E71.10 of this study was placed to *Vischeria*, but no species assignation was possible and no species description was attempted in this study. A better resolution for the genetic distinction of species within *Vischeria* clade was achieved using ITS2 rDNA marker. However, the presence of multiple paralogous sequences within one strain, i.e., an intragenomic ITS2 sequence variation, was revealed for Eustigmatophyceae (Kryvenda et al. 2018), which makes further taxonomic assignments among investigated *Vischeria* strains impossible, until the divergence of intragenomic ITS2 paralogues in this genus is better understood.

### Biomass, fatty, and amino acid yield

The faster growth and productivity rates were achieved with 3 N BBM and elevated amounts of CO_2_, at almost 0.2 g per liter reactor volume per day, which is in accordance with many other studies (e.g., Wang et al. 2018). To increase the yield of fatty acids, the logarithmic growth phase should be followed by exposure to sodium chloride and nitrogen depletion before harvest. Wang et al. (2018) found high amount of palmitoleic acid (C16:1) in several Eustigmatophyceae, ranging from 15% DM to approx. 25% DM under decreasing nitrogen availability. Here, this FA was the second most abundant, respectively, most dominant FA in case of -N BBM, and with comparable amounts to the reference. However, the yield was lower when applying 3 N BBM without additional CO_2_ and worst under sodium chloride stress reaching only about 5% DW. Remarkable are the high contents of the valuable EPA; however, its concentration was not elevated by application of nitrogen stress. Except for the treatment with -NBBM, the EPA content was higher (expressed as % in total fatty acids) by 10 to 20% when compared to for ITS2 rDNA almost genetically identical strain *Vischeria* sp. SAG 51.91 (Lang et al. 2011). In the eustigmatophycean alga *Trachydiscus guangdongensis*, EPA was also the most prominent FA, but constantly decreased during cultivation (Gao et al. 2019). In a study of Cepák et al. (2014) with the same species, EPA content was highest at the lowest light level they applied (100 μmol photosynthetic active radiation (PAR)/m^2^/s). In *Vischeria* sp. E71.10, EPA was produced fastest in high nitrogen medium and with additional application of CO_2_. The production rate of 32.32 mg/L/day was extraordinarily high compared to other studies involving Eustigmatophyceae. Qunju et al. (2016) reported only a maximum of 9.64 mg/L/day for total poly unsaturated fatty acid yield of a high productivity strain, *Nannochloropsis* sp.

The applied HPLC method proofed to be both economic and feasible for the determination of algal amino acids. Chua and Schenk (2017) reviewed methods to economically harvest proteins from Eustigmatophyceae used as an alternative source for animal feed and foodstuff. Generally, studies about individual AA spectra are sparse. Tibbetts et al. (2015) is one of the few studies analyzing a Eustigmatophyceae, *Nannochloropsis granulata*, and like in this study, glutamic acid was the most abundant AA.

## Conclusions

The molecular ITS2 rDNA marker was useful for placing strain into E71.10 to *Vischeria*. The culture performed well in terms of high growth rates and abundant fatty acid accumulation, namely EPA, confirming the biotechnological potential and oleaginous character of such eustigmatophycean microalgae. Moreover, old cultures turning from green to orange indicate carotenoid accumulation during nitrogen starvation. These facts underline the potential of this strain as a candidate for sustainable bioresources. Further investigations should include an optimization of the reactor settings for enhanced yield and testing the pigment (carotenoid) production as an additional valuable biocompound.

## Electronic supplementary material

ESM 1 (DOCX 63 kb)

ESM 2 (AVI 1256638 kb)
