# Supplementary material for: Growth, fatty, and amino acid profiles of the soil alga Vischeria sp. E71.10 (Eustigmatophyceae) under different cultivation conditions
Source: Folia Microbiol (Praha). 2020 Jul 21;65(6):1017–23. doi: 10.1007/s12223-020-00810-8 (PMC7716935; doi:10.1007/s12223-020-00810-8)
Supplement: Supplementary file 1 — (DOCX 63 kb) [file 12223_2020_810_MOESM1_ESM.docx]

## **Electronic supplementary material**

**Growth, fatty- and amino-acid profiles of the soil alga *Vischeria* sp. E71.10 (Eustigmatophyceae) under different cultivation conditions**

**Daniel Remias^1*^, Cecilia Nicoletti^1^, Klaus Krennhuber^1^, Bettina Möderndorfer^1^, Linda Nedbalová^2^, Lenka Procházková^2^**

^1^University of Applied Sciences Upper Austria, School of Engineering, 4600 Wels, Austria

^2^Charles University, Faculty of Science, Department of Ecology, 12843 Prague, Czech Republic

*Corresponding author: [daniel.remias@fh-wels.at](mailto:daniel.remias@fh-wels.at), Orcid: 0000-0003-0896-435X

**Supplementary Video** (E71.10_flagellate.avi) **s**howing the rotary movement of one rare flagellate cell among immotile stages of *Vischeria* sp. E71.10. Duration 1 min 24 sec

**
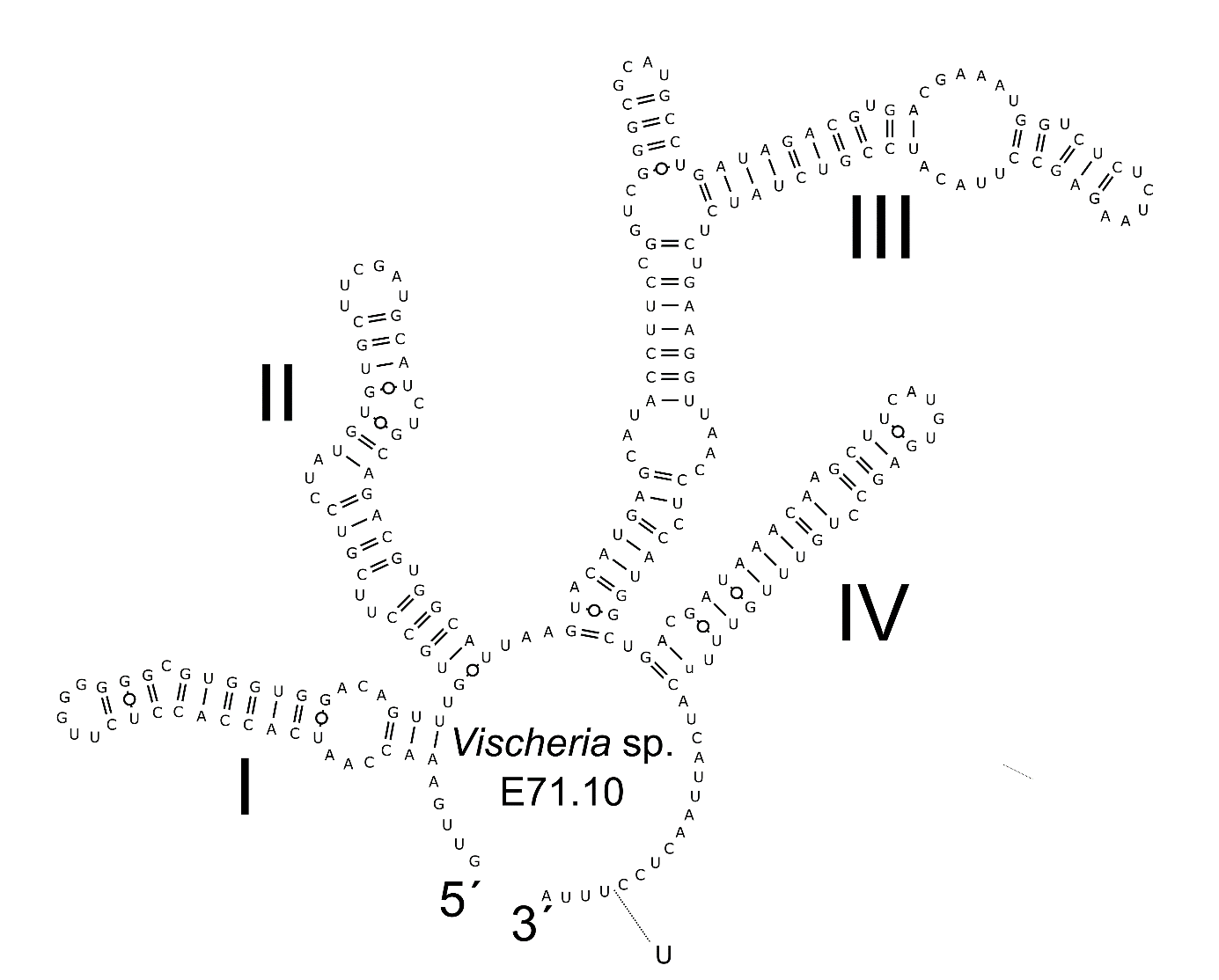
**

**Supplementary Figure 1** Comparison of the ITS2 rRNA secondary structure of strains *Vischeria* sp. E71.10 (MN781106) and *Vischeria* sp. SAG 51.91 (formerly assigned to *Chloridella simplex*; KY271671.1). Helices are labeled with Latin numbers: I–IV. A single indel between both strains is indicated outside the structure and linked by a dotted line**.**

**Supplementary Table S1** List of primers used for amplification of 18S rDNA (18S), ITS1 rDNA, ITS2 rDNA (ITS) and *rbc*L markers; (F) forward; (R) reverse

| Primer | Marker | Direction | Sequence | Reference |  |
| --- | --- | --- | --- | --- | --- |
| P2 | 18S | F | CTGGTTGATTCTGCCAGT | Wever et al. (2009) | |
| P4 | 18S | R | TGATCCTTCYGCAGGTTCAC | Moon-van der Stay et al. (2000) | |
| ITS-F-Visch | ITS | F | GCGCCGTTGGCTTCTAGCC | M. Eliáš, pers. comm. | |
| ITS4k-Eustig | ITS | R | TCCTCCGCTTAGTTATATGC | M. Eliáš, pers. comm. | |
| eustig-*rbc*LF | *rbc*L | F | GATCCRATTGAAGCTGC | Prior et al. (2009) | |
| DP*rbc*L7 | *rbc*L | R | AAASHDCCTTGTGTWAGTYTC | Daugbjerg and Andersen (1997) | |

**Supplementary Table S2:** *Vischeria* sp. (E71.10) blast search results for the three molecular marker regions. Length of the base pair sequence, percentage of sequence identity and NCBI accession number are indicated.

a) **18S rDNA** (1595 bp)

| 100 % identical *Vischeria polyphem* strain CAUP Q 102 KF848922.1 |
| --- |
| 100 % identical *Eustigmatos polyphem* isolate CCAP 860/8 MG022744.1 |
| 100 % identical *Eustigmatos magnus* strain SAG 2266 KY271669.1 |
| 100 % identical *Vischeria helvetica* strain CCALA 514 KF848920.1 |
| 100 % identical *Vischeria helvetica* KGU-Y001 AB731568.1 |
| 100 % identical *Vischeria helvetica* voucher UTEX 49 HQ710569.1 |
| 100 % identical *Vischeria helvetica* strain CCALA 514 KF848920.1 |
| 100 % identical *Chloridella simplex* strain CCALA 279 KF848923.1 |
| 100 % identical *Vischeria* sp. IPPAS C-70 MN164434.1 |
| 100 % identical *Vischeria* sp. YACCYB463 MH683913.1 |
| 100 % identical *Vischeria* sp. YACCYB461 MH683912.1 |
| 100 % identical *Vischeria* sp. YACCYB423 MH683892.1 |

1. ***rbc*L** (1248 bp)

| 99 % - 1 bp difference - *Vischeria helvetica* voucher UTEX 49 HQ710612.1 |
| --- |
| 99 % - 6 bp difference - *Eustigmatos magnus* strain SAG 2266 KY271708.1 |
| 99 % - 8 bp difference - *Vischeria* sp. strain CAUP Q 202 KX839261.1 |

For the other abovementioned strains - which were identical for 18S rDNA marker – the *rbc*L sequence is not available.

1. **ITS1 + 5.8S + ITS2 rDNA** (678 bp)

| 99 % identical - ***Vischeria* sp.** strain SAG 51.91 KY271671.1 |
| --- |
| 1 bp difference in ITS1 rDNA |
| 1 bp difference in 5.8S rDNA |
| 1 indel in ITS2 (at the end of sequence, could be a sequencing error) |

For the other abovementioned strains, which had identical 18S rDNA, the region of the ITS2 rDNA is shown for comparison:

| 97 % identical – 8 bp difference and 5 indels - *Eustigmatos magnus* strain SAG 2266 KY271676.1 |
| --- |
| 96 % identical – 4 bp difference and 8 indels - *Vischeria* sp. IPPAS C-70 MN164431.1 |

**Supplementary References**

Daugbjerg N, Andersen, R.A (1997) A molecular phylogeny of the heterokont algae based on analyses of chloroplast‐encoded rbcL sequence data 1. J Phycol 33(6): 1031–1041. <https://doi.org/10.1111/j.0022-3646.1997.01031.x>

De Wever A, Leliaert F, Verleyen E, Vanormelingen P, Van der Gucht K, Hodgson DA, Sabbe, K, Vyverman W (2009) Hidden levels of phylodiversity in Antarctic green algae: further evidence for the existence of glacial refugia. P Roy Soc B-Biol Sci 276(1673): 3591–3599. <https://doi.org/10.1098/rspb.2009.0994>

Moon‐van der Staay SY, van der Staay GW, Guillou L, Vaulot D, Claustre H, Medlin LK (2000) Abundance and diversity of prymnesiophytes in the picoplankton coμmunity from the equatorial Pacific Ocean inferred from 18S rDNA sequences. Limnol Oceanogr 45(1): 98–109. <https://doi.org/10.4319/lo.2000.45.1.0098>

Prior SE, Fawley MW, Fawley KP (2009) DNA sequence analysis of freshwater Eustigmatophyceae, a potential source of essential fatty acids. J Ark Acad Sci 63(1): 139–144. <https://scholarworks.uark.edu/jaas/vol63/iss1/17/>
